# TRIM9 Interacts with ZEB1 to Suppress Esophageal Cancer by Promoting ZEB1 Protein Degradation via the UPP Pathway

**DOI:** 10.1155/2023/2942402

**Published:** 2023-04-20

**Authors:** Zhaoxian Lin, Jianyuan Huang, Lihuan Zhu, Xing Lin, Yangyun Huang, Chun Chen, Xiaojie Pan

**Affiliations:** ^1^Department of Thoracic Surgery, Shengli Clinical Medical College of Fujian Medical University, Fujian Provincial Hospital, No. 134, East Street, Fuzhou 350001, China; ^2^Department of Thoracic Surgery, Fujian Medical University Union Hospital, No. 29, Xinquan Road, Gulou District, Fuzhou 350001, China

## Abstract

**Background:**

Esophageal cancer remains one of the most lethal malignant diseases globally. Previous studies indicated that TRIM9 (Tripartite Motif Containing 9) is a potential marker in breast cancer patients. Therefore, in the current research, we intended to clarify the regulatory network of TRIM9 and its relative role in esophageal cancer patients. We aimed to elucidate the regulatory role of TRIM9 in esophageal cancer.

**Methods:**

Clinical tumor tissue samples combined with cancer cell line models were utilized to explore the TRIM9 expression pattern. Functional experiments including transwell assay, cell viability assay, and ubiquitination blocking experiments were performed to evaluate the role of the TRIM9/ZEB1 (zinc finger E-box binding homeobox 1) axis and UPP pathway in esophageal cancer progression and exacerbation.

**Results:**

Both esophageal cancer samples and cell line models showed significantly suppressed levels of TRIM9. Functional experiments confirmed that TRIM9 overexpression inhibited the cell viability, invasiveness, and stem-like phenotype of cancer cells. Subsequent investigations suggested that TRIM9-ZEB1 interaction accelerated ZEB1 protein degradation through the modulation of the UPP pathway, which confirmed the protective role of TRIM9 in esophageal cancer progression and metastasis.

**Conclusion:**

This study concluded that TRIM9 was a tumor suppressor that interacted with ZEB1 and accelerated ZEB1 protein degradation via the ubiquitin-proteasome pathway (UPP). Our research emphasized TRIM9-ZEB1 interaction as a valuable target for esophageal cancer treatment in future development. More detailed studies are needed to further consolidate our findings.

## 1. Introduction

Currently, esophageal cancer remains one of the top 10 lethal diseases in overall mortality [1]. Based on the statistics, the majority of the cases occur in males and the gender and region differences in esophageal cancer incidences are quite obvious. Esophageal cancer is most commonly seen in Eastern and Southern African countries. It has been suggested that drinking and smoking are important risk factors for the pathogenesis of squamous cell carcinoma. For early-stage patients, endoscopic resection (ER) combined with chemoradiotherapy (CRT) is an effective strategy. As for advanced-stage patients, multidisciplinary treatment combined with surgery and immunotherapy is reshaping the picture of long-term prognosis for this group of patients [2]. However, refractory and metastatic diseases are one of the major threats for esophageal cancer patients. Therefore, current investigators aim to clarify the mechanism of esophageal cancer refractoriness and metastasis so as to overcome the barriers against superior therapeutic effects and patient prognosis.

Epithelial-mesenchymal transition (EMT) is a crucial process that cancer cells exploit for metastasis by transition of their epithelial phenotype into a mesenchymal phenotype. Previous research studies have confirmed that EMT not only participates in the carcinogenesis and progression of malignancies but also plays a unique role in cancer metastasis and chemorefractoriness [3–5]. *In vitro* analysis of an immunodeficient mouse model also indicated EMT was involved in the regulation of tumors, which greatly enhanced tumor cell self-renewal and differentiation [6–8].

The ubiquitin-proteasome system is a vital regulator of cell protein homeostasis [9–12]. The ubiquitin proteolysis regulatory pathway includes several constituents including E1 activating enzymes, E2 conjugating enzymes, and E3 ubiquitin ligases. Previous studies on RING and HECT family E3 ligases indicated that they tightly regulated the EMT process through ubiquitination [13–17]. The tripartite motif protein (TRIM) family also belongs to the group of E3 ubiquitin ligases. TRIM9, as a component of the TRIM family, has been demonstrated as a potential biomarker for breast cancer detection [18]. However, the exact role of TRIM9 in esophageal cancer exacerbation and metastasis is not clarified. Therefore, we aimed to investigate the significance of TRIM9 and its related molecular regulatory network in esophageal cancer.

Zinc finger E-box binding homeobox 1 (ZEB1) has been proven to be closely related to progression and tumors. ZEB1 promotes the Warburg effect to increase tumorigenesis and metastasis of hepatocellular carcinoma by transcriptionally activating PFKM [19]. The ZEB1 transcription factor was identified as a key determinant of melanoma immune escape [20]. ZEB1 contributed greatly to the EMT process of tumor metastasis [21]. However, whether TRIM9 could regulate esophageal cancer by affecting ZEB1 remains unclear.

## 2. Materials and Methods

### 2.1. Patient Recruitment and Sample Collection

Patients diagnosed with esophageal cancer by their physicians were enrolled in this study. Biopsy samples were harvested prior to any surgical, radiological, or chemotherapy treatment. Esophageal tumor samples as well as matched surrounding normal tissues were simultaneously collected. All samples were immediately transported to the lab for the following assays. The Declaration of Helsinki was followed in this study. All informed consent forms of each enrolled individual were completed and obtained.

### 2.2. Vector Construction and Transfection

pLVX vectors were purchased from Clontech (#632164, Takara, CA, USA). Polymerase chain reaction (PCR) was conducted using DNA template sequences. PCR products were first purified using 1% agarose gel and double digested by BamHI and EcoRI along with empty pLVX vectors. A ligation reaction was conducted overnight between the vectors and the purified PCR products using T4DNA ligase. A ligation product was further used for the *E. coli* DH5*α* competent cell transformation. Cell clones were then added to the ampicillin-containing LB plate and were treated overnight at 37°C. Positive clones were collected, and the plasmid was extracted and then sequenced (Shanghai Invitrogen Biotech Co., Ltd). Then, the lentiviral vectors were packaged and subsequently added into tumor cell groups with a multiplicity of infection (MOI) of 20, and the vector titer was set as 1.5 × 10^9^/ml. Subsequently, the fluorescent protein expression level and transfection efficiency were assessed 1-2 days after transfection.

### 2.3. RNA Extraction and qRT-PCR Experiments

Total RNA was extracted from cells by using the TRIzol reagent (Invitrogen, Carlsbad, USA). To synthesize the complementary DNA, oligo-dT primers and reverse transcriptase (Takara) were used in the mixture of reaction buffer with each sample according to the kit instructions by the manufacturer. qRT-PCR was performed using the SYBR Green qPCR Mix Kit (Genstar) and the primers listed in supplementary table 1. Quantification statistics which were calculated using the cycle threshold method were finally normalized according to the cycle threshold (CT) value of GAPDH, and the relative level of gene expression was obtained.

### 2.4. Cell Line Culture

KYSE-150, KYSE-30, TE-10, TE-11, TE-1, CaES-17, NEC, TE-13, KYSE-410, and Het-1A cells were stored in our institutional research center and used for this study. Cells were incubated in DMEM containing 10% fetal bovine serum (FBS), antibiotics (100 IU penicillin and 100 *μ*g/ml streptomycin), and growth supplement at the condition of 5% CO_2_ at 37°C. The above cell types were incubated in tissue culture plastic flasks or 24-well plates. At confluence, to perform cell passage, trypsin digestion for 2 minutes was conducted. For statistical intent, the density of cell seeding was kept at approximately 40,000 cells/cm^2^ for the following assays.

### 2.5. Immunoprecipitation and Immunoblot

After appropriate treatments, the cells were rinsed with PBS three times and collected. Then, the immunoprecipitation lysis buffer containing protease and phosphatase inhibitor cocktail was used for cell sample lysis. Cell lysates then underwent centrifugation and preclearing. Supernatants were incubated with appropriate antibodies (5 *μ*g anti-FLAG and A/G Sepharose beads) at 4°C overnight. Immunoprecipitates were obtained after washing three times. The immunoprecipitates and inputs were analyzed by the Western blot procedure as described previously elsewhere.

### 2.6. Cell Growth Test

To assess cell growth, 1000 cells were seeded in 6-well plates for each group. After appropriate treatment such as vector transfection, absorbance was recorded at 490 nm by a microplate reader.

### 2.7. Migration/Invasion Assay

For the transwell study, 1 × 10^6^ tumor cells from each cell group were first seeded in the upper chamber with a matrix gel (1 : 6 diluted, Corning, ME) with FBS-free DMEM. Subsequently, DMEM containing 10% FBS was placed in the lower chamber for chemoattraction. Mitomycin C was further added to the upper chamber to stop cell proliferation. After 24 h of incubation, the fraction of tumor cells that invaded the lower surface of the membrane was fixed using 4% methanol, treated with crystal violet staining, and subsequently quantified in 15 random ×100 microscopic fields per sample.

### 2.8. Wound Healing Assay

As for the wound healing experiment, each group of cells was placed and monolayers were scraped. The distance between wounds was tested three times. The distance between the wounds was repeatedly measured after incubation for 48 h.

### 2.9. Immunofluorescence (IF) Staining

Firstly, cell samples were washed and fixed with 4% paraformaldehyde. After permeabilization with 0.3% Triton X-100 in TBS for 15 minutes, samples were blocked with 1% BSA in PBS for 90 minutes. The cells were further incubated with appropriate primary antibodies at 4°C overnight. Subsequently, the cells were incubated with conjugated secondary antibodies for 1.5 h. Then, the nuclei were stained by DAPI (blue). The immunofluorescence images of samples were captured by confocal microscopy for image generation. Three independent pathologists evaluated the immunohistochemical staining results.

### 2.10. Ubiquitination Test

The ubiquitination test was performed based on the method described previously [22]. Firstly, cells were treated with MG132 for 6 hours before harvesting for transfection. Afterwards, the cell lysate was lysed by lysis buffer. The proteins in the cell groups were then immunoprecipitated (IP) with the indicated antibodies. The ubiquitination of cell proteins was investigated and tested by Western blotting.

### 2.11. Western Blotting

The BCA method was applied to measure protein concentration. SDS-PAGE was performed to separate proteins, and samples were transferred to a PVDF membrane (Sigma, USA). After blocking with TBST containing 5% nonfat milk, the membranes were incubated with primary antibodies and secondary antibodies successively. Finally, an enhanced chemiluminescence detection kit (Thermo Fisher, USA) was used to detect target proteins, and ImageJ software was used to observe protein bands.

### 2.12. Statistical Analysis

Data were statistically analyzed using SPSS (version 19). Data were collected from experiments that were repeated three times. Significance was set at *p* value less than 0.05. The *t*-test and ANOVA test were used for *p* value determination. Error bars indicate SD. Asterisks in the figures indicate significant statistical differences between the compared groups.

## 3. Results

### 3.1. TRIM9 Expression Was Repressed in Tumor Cells and Correlated with Worse Prognosis for Esophageal Cancer Patients

**Figure 1:**
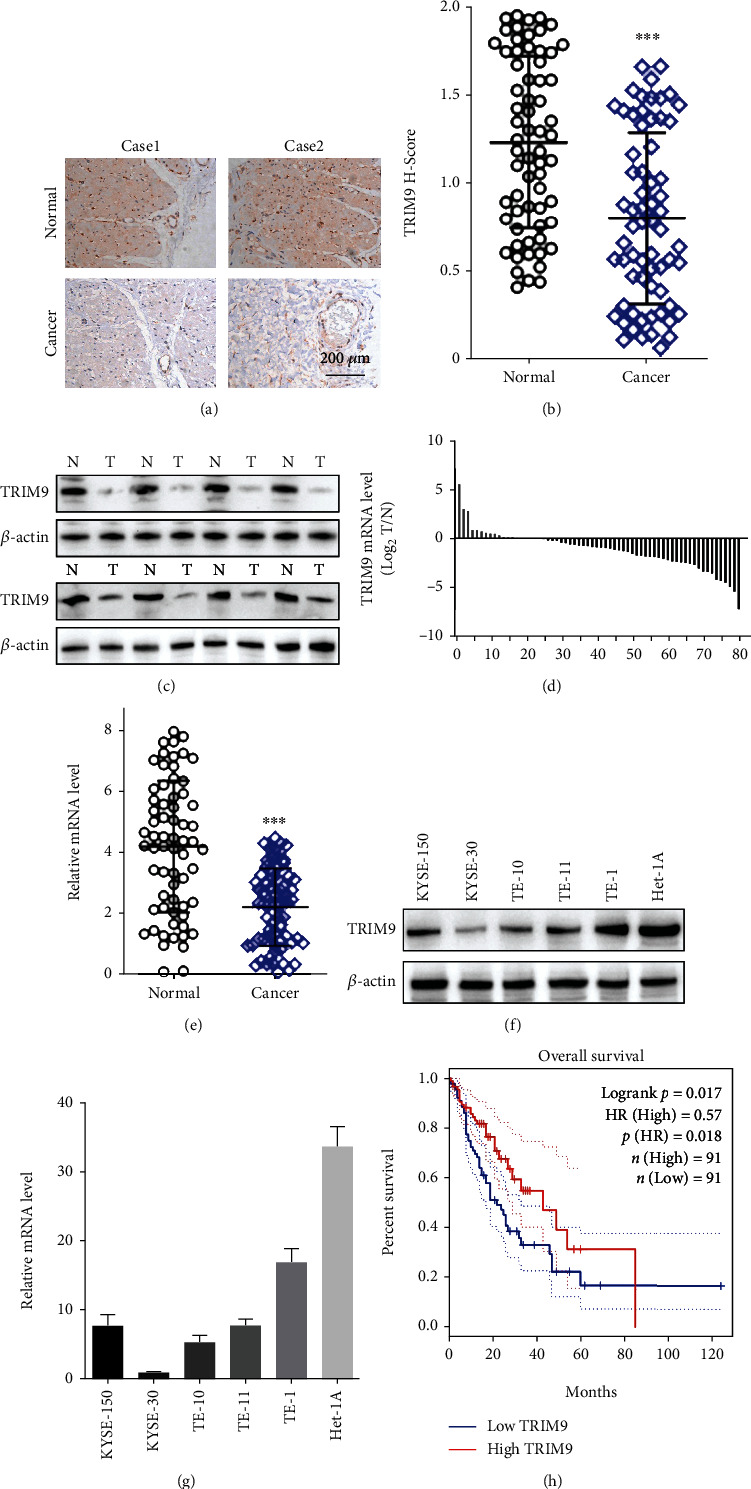
TRIM9 expression was repressed in tumor cells and correlated with worse prognosis for esophageal cancer patients. (a, b) Expression of TRIM9 protein in esophageal tumor tissue and adjacent matched normal tissue samples detected by IHC assay and TRIM9 *H*-score comparison between clinical samples of the esophageal cancer cohort. (c) WB analysis of TRIM9 protein expression among four matched samples of esophageal cancer tumor tissue and surrounding normal tissue. (d) TRIM9 mRNA expression ratio between esophageal cancer tumor tissue and matched adjacent normal tissue. Postsurgical samples were gathered from esophageal cancer patients in our clinical center. (e) TRIM9 mRNA level comparison between esophageal cancer tissue and matched adjacent surrounding tissue collected in our clinical center. (f, g) TRIM9 protein and mRNA levels detected by WB and quantitative RT-PCR experiments in several esophageal cancer cell lines including KYSE-150, KYSE-30, CaES-17, TE-10, NEC, TE-11, TE-1, TE-13, and KYSE-410. (h) Kaplan-Meier plot and log-rank test were utilized to evaluate the overall prognosis of esophageal cancer patients. Two patient groups were divided based on the expression of TRIM9 gene expression compared with the median level.

In order to investigate the protein expression of TRIM9 in esophageal cancer cells, IHC slides of esophageal cancer samples and corresponding adjacent normal samples were utilized. As shown in Figure 1(a), TRIM9 expression was significantly decreased in esophageal tumor tissue. *H*-score comparison between esophageal cancer samples and matched normal samples also confirmed the above findings (Figure 1(b)). WB analysis of 4 pairs of matched tumor and normal tissues also suggested notably suppressed TRIM9 protein levels in tumor samples (Figure 1(c)). In addition, PCR experiments on clinical tumor samples also demonstrated significantly depressed TRIM9 mRNA expression in the majority of tumor tissue (Figures 1(d) and 1(e)). Consistent with our findings, TRIM9 mRNA as well as the protein expression pattern was further validated in esophageal cancer cell lines/normal human epithelial cell lines. Results indicated that the majority of cancer cell lines exhibited significantly lower mRNA/protein levels compared with normal esophageal epithelial cell lines (Figures 1(f) and 1(g)). Moreover, prognosis evaluation of patient groups with low/high TRIM9 expression indicated that high TRIM9 expression levels were associated with superior overall survival, in contrast with their low TRIM9 counterparts (*p* = 0.017) (Figure 1(h)).

### 3.2. TRIM9 Suppression Aggravates Malignancy of Esophageal Cancer Cells

**Figure 2:**
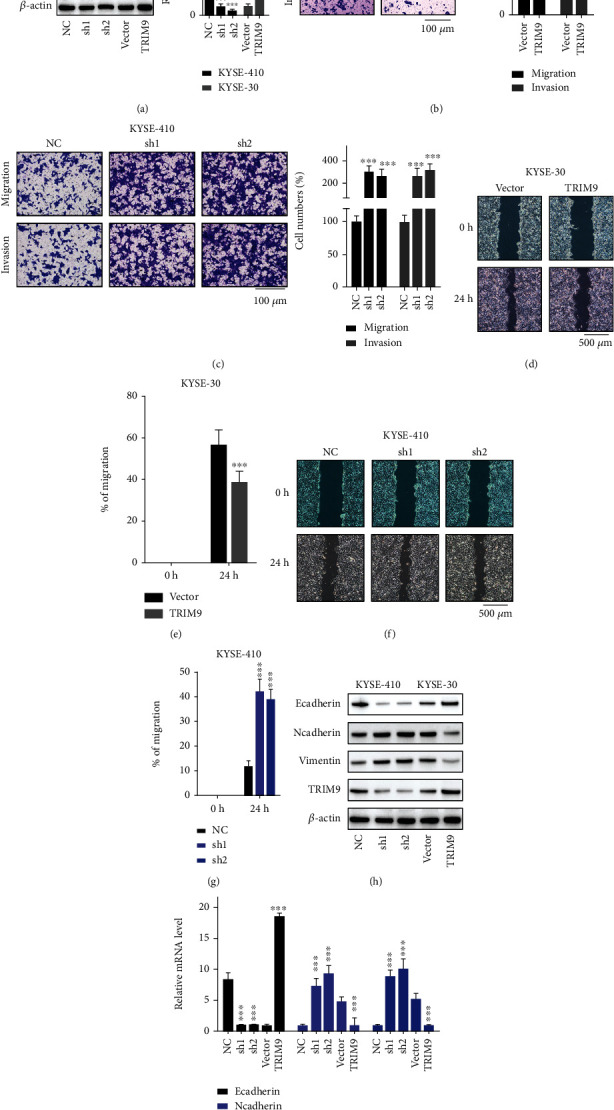
TRIM9 suppression aggravates malignancy of esophageal cancer cells. (a) mRNA and protein expression of TRIM9 modulated by TRIM9-specific shRNAs (sh1/sh2) and TRIM9 overexpression plasmid in KYSE-410 and KYSE-30 esophageal cancer cell lines. (b, c) Functional tests on tumor cell migration and invasion capability of KYSE-30/KYSE-410 cell lines transfected with TRIM9 overexpression vectors or TRIM9-specific shRNAs. Percentage of cell migration and invasion of each cell group was calculated and statistically compared. (d–g) Wound healing experiment on KYSE-30/KYSE-410 cell lines transfected with TRIM9-specific overexpression vectors and TRIM9-specific shRNAs, respectively. Percentage of migrated tumor cells in the two cell groups was further calculated and statistically compared. (h, i) Protein and mRNA EMT biomarkers (E-cadherin, N-cadherin, and vimentin) were detected by WB/qRT-PCR experiments. Tumor cell group was transfected with TRIM9-specific shRNAs or overexpression vectors.

To understand the functional impact of TRIM9 expression on esophageal cancer cells, TRIM9 shRNAs as well as overexpression vectors were used to modulate TRIM9 expression in the esophageal cancer tumor cell lines KYSE-30 and KYSE-410 (Figure 2(a)). Then, transwell and wound healing assays were conducted and results showed that TRIM9 overexpression obviously reduced KYSE-30 cell migration and invasion significantly (Figures 2(b), 2(d), and 2(e)). Meanwhile, TRIM9 shRNA-treated KYSE-410 tumor cells demonstrated augmented mobility and invasion (Figures 2(c), 2(f), and 2(g)). Subsequent molecular experiments showed that TRIM9 suppression was associated with the enhanced mesenchymal phenotypic transition of tumor cells, since the mRNA and protein expression of mesenchymal-related cell biomarkers (N-cadherin and vimentin) was significantly increased in the TRIM9 shRNA-treated KYSE-410 cell group, while the expression of an epithelial-related biomarker (E-cadherin) was suppressed (Figures 2(h) and 2(i)).

### 3.3. Influence of TRIM9 on Chemoresistance of KYSE-410 Cells

**Figure 3:**
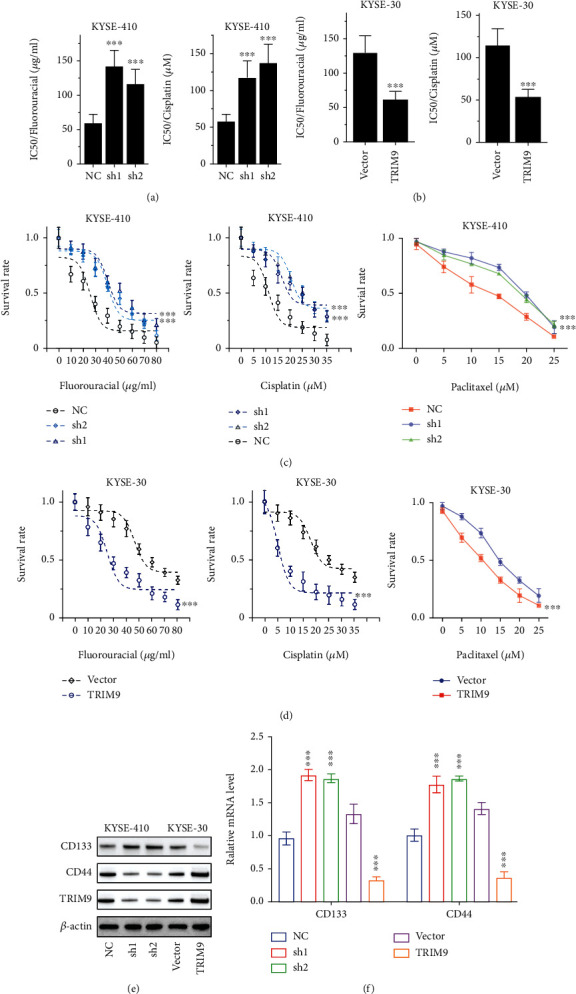
Influence of TRIM9 on chemoresistance of KYSE-410 cells. (a, b) IC50 level detection of chemoagents including fluorouracil and cisplatin treated on esophageal cancer cells. KYSE-410 and KYSE-30 cell line groups were treated with TRIM9-specific shRNAs and overexpression vectors. (c, d) Chemoresistance evaluation of KYSE-30/KYSE-410 cell line groups under the treatment of 5-FU, cisplatin, or paclitaxel. Cancer cells in each of the groups were separately transfected with TRIM9-specific shRNAs and overexpression vectors/control vectors. (e, f) WB/quantitative RT-PCR detection of cancer stem-like cell biomarker (CD133/CD44) levels. Tumor cells in each group were separately treated with TRIM9-specific shRNAs or TRIM9-specific overexpression vectors and negative control vectors.

Furthermore, cell viability tests using 5-FU and cisplatin indicated that TRIM9 silencing significantly increased the chemoresistance of KYSE-410 cells, as the IC50 of shRNA-treated cell groups notably increased (Figure 3(a)). In contrast, KYSE-30 cells treated with TRIM9 overexpression vectors exhibited significantly lower levels of IC50, suggesting notably alleviated chemoresistance of the tumor cells (Figure 3(b)). Consistently, cell survival assays on KYSE-410 and KYSE-30 cell groups under the treatment of 5-FU, cisplatin, or paclitaxel indicated that TRIM9 shRNA-treated tumor cells exhibited a significantly increased survival rate compared with the control group (Figure 3(c)), whereas TRIM9 overexpression vector-transfected tumor cells demonstrated notably decreased cell survival (Figure 3(d)). Besides, subsequent exploration suggested that TRIM9 shRNA treatment resulted in increased mRNA and protein expression of stem-like phenotypic biomarkers on tumor cells, including CD133 and CD44, whereas TRIM9 overexpression demonstrated completely opposite effects (Figures 3(e) and 3(f)).

### 3.4. TRIM9 Inhibits Esophageal Cancer Cell Migration and Invasion by Regulating ZEB1 via the Ubiquitin-Proteasome Pathway

**Figure 4:**
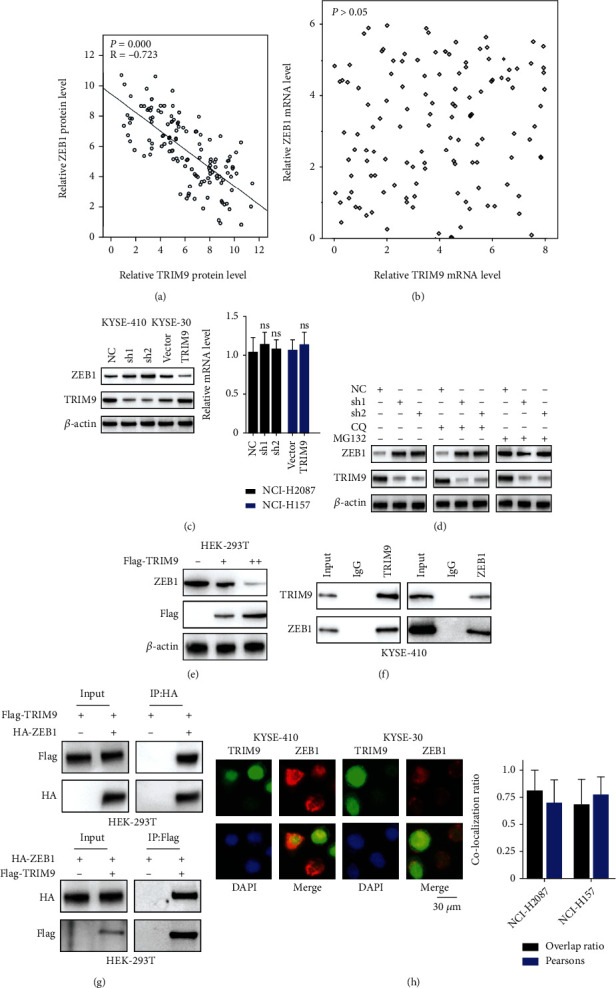
TRIM9 inhibits esophageal cancer cell migration and invasion by regulating ZEB1 via the ubiquitin-proteasome pathway. (a, b) Association of the ZEB1 and TRIM9 protein or mRNA expression levels in esophageal cancer samples. Linear regression analysis was performed to examine statistical significance. (c) WB and quantitative RT-PCR detection of ZEB1 protein and mRNA levels in KYSE-410 and KYSE-30 cell lines. Each group of cancer cells was separately transfected with TRIM9-specific shRNAs or TRIM9-specific overexpression vectors and negative control vectors. (d) TRIM9 and ZEB1 protein level detection via WB experiments on esophageal cancer cell groups separately transfected with TRIM9-specific overexpression vectors or control vectors, in combination with or without treatment of UPP inhibitors (CQ and MG132). (e) Protein level quantification by WB experiments of FLAG-tagged TRIM9 and ZEB1 in HEK-293T cells. (f, g) Coimmunoprecipitation assay on the interaction of TRIM9 with ZEB1 in KYSE-410 and HEK-293T cell lines. (h) Immunofluorescence assay and colocalization ratio quantification were performed to investigate the colocalization of TRIM9 and ZEB1 in KYSE-30 and KYSE-410 cell lines.

Next, in order to unveil the underlying molecular mechanism of TRIM9's impact on esophageal cancer, we further explore the expression level association between ZEB1 and TRIM9. Results from the TRIM9 mRNA and protein quantification study of esophageal cancer samples suggested that ZEB1 protein levels were significantly correlated with TRIM9 (*p* < 0.001), but for their mRNA expression levels, no notable correlation was detected (Figures 4(a) and 4(b)). Subsequently, utilizing cell line models, we also demonstrated that TRIM9 modulation showed no impact on ZEB1 mRNA expression but exhibited a significant modulatory effect on ZEB1 protein expression (Figure 4(c)), which indicated the posttranscriptional regulatory function of TRIM9 on ZEB1. Therefore, we further applied an autophagy inhibitor (CQ) and a proteasome inhibitor (MG132) to explore the role of autophagy and the ubiquitin-proteasome pathway (UPP) in ZEB1 expression modulation. Results indicated that TRIM9 overexpression tumor cells treated with or without CQ exhibited decreased ZEB1 protein levels, whereas MG132-treated tumor cells demonstrated no such effects (Figure 4(d)). In addition, we also demonstrated using HEK-293T as a model that FLAG-TRIM9 transfection caused ZEB1 protein expression inhibition in a dose-dependent manner (Figure 4(e)). Furthermore, we utilized coimmunoprecipitation assay and immunofluorescence colocalization assay to provide consolidation evidence that TRIM9-ZEB1 interaction occurred in KYSE-410 and KYSE-30 cells (Figures 4(f)–4(h)).

### 3.5. Influence of TRIM9 on ZEB1 Degradation Velocity in KYSE-410 Cells

**Figure 5:**
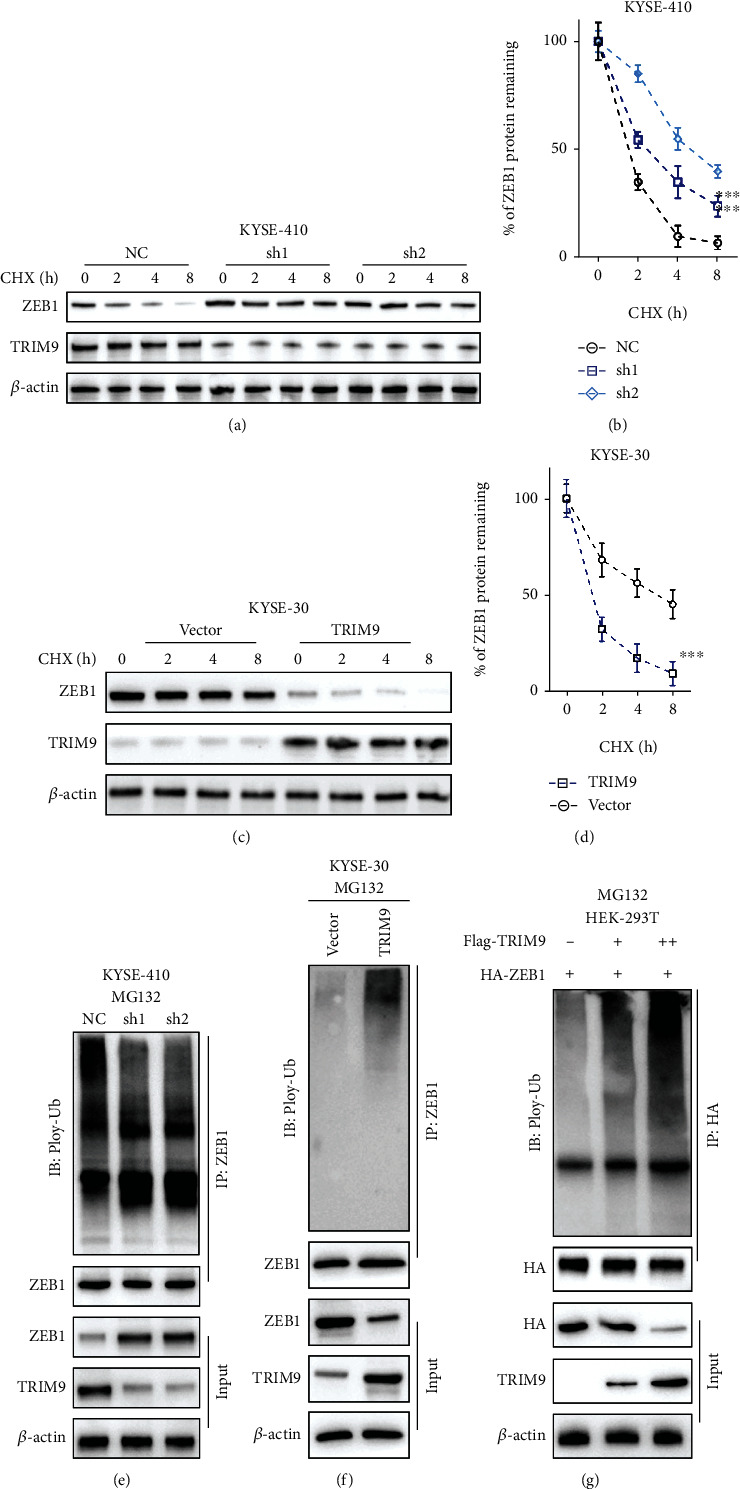
Influence of TRIM9 on ZEB1 degradation velocity in KYSE-410 cells. (a–d) CHX chase assay to evaluate the influence of UPP blockage on TRIM9 and ZEB1 expression in KYSE-410 (a, b) and KYSE-30 (c, d) cell lines. Each cancer cell group was separately transfected with TRIM9-specific shRNAs or TRIM9-specific negative control vectors. (e, f) Co-IP assay to evaluate the impact of TRIM9 expression regulation on ZEB1 protein levels in esophageal cancer cell lines (KYSE-410 and KYSE-30). (g) Each group of tumor cells was treated with MG132 to block the UPP pathway and was synergistically transfected with TRIM9-specific shRNAs or TRIM9-specific overexpression vectors.

Besides, cycloheximide (CHX) chase assay was also applied to investigate protein degradation velocity in KYSE-410 cells treated with TRIM9 shRNAs or KYSE-30 cells treated with TRIM9 overexpression vectors. For the KYSE-410 cell group treated with TRIM9 shRNAs, protein degradation of ZEB1 was significantly slower (Figures 5(a) and 5(b)). In contrast, for KYSE-30 cells treated with TRIM9 overexpression vectors, ZEB1 protein degradation velocity drastically increased (Figures 5(c) and 5(d)). Consistently, co-IP experiments also demonstrated that for KYSE-410 and KYSE-30 under the treatment of MG132, the TRIM9 shRNA-treated KYSE-410 cell group exhibited notably reduced ubiquitination of ZEB1, resulting in increased ZEB1 protein levels (Figure 5(e)). However, KYSE-30 cells treated with TRIM9 overexpression vectors drastically promoted ZEB1 ubiquitination, and decreased ZEB1 protein levels were witnessed (Figure 5(f)).

### 3.6. Influence of ZEB1 on the Malignant Phenotype of Esophageal Cancer Cells

**Figure 6:**
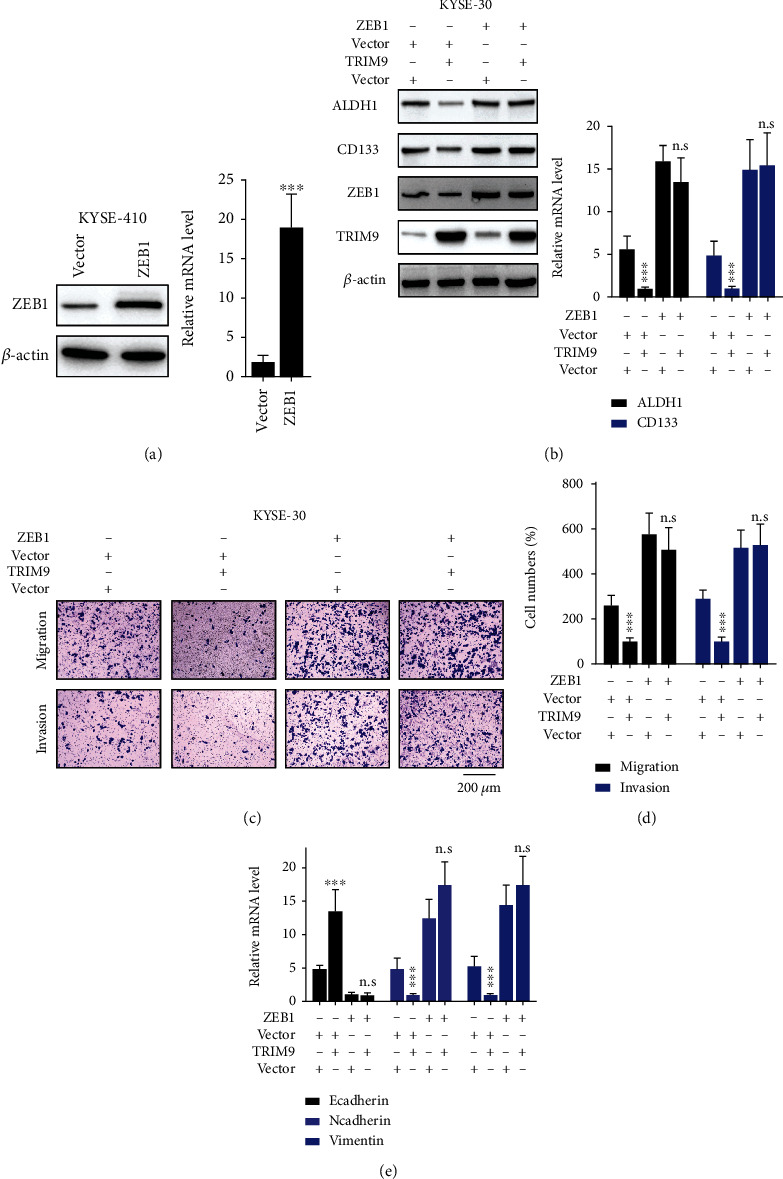
Influence of ZEB1 on the malignant phenotype of esophageal cancer cells. (a) WB and quantitative RT-PCR assay to evaluate the effects of ZEB1-specific overexpression vectors on ZEB1 protein and mRNA levels. (b) WB and quantitative RT-PCR experiments to evaluate cancer stem-like cell biomarkers including ALDH1 and CD133 in KYSE-30 tumor cells. Each group of cells was separately transfected with ZEB1-specific overexpression vectors in combination with or without TRIM9-specific overexpression vectors. (c, d) Transwell assay to investigate the effects of TRIM9-specific overexpression vectors combined with or without ZEB1-specific overexpression vector transfection on KYSE-30 cancer cell migration and invasion. Percentage of migrated cells for each group was quantified and statistically compared. (e) Quantitative RT-PCR evaluation of the mRNA expression of EMT-associated biomarkers including E-cadherin, N-cadherin, and vimentin in esophageal cancer cell lines. Each cell group was separately transfected with TRIM9-specific overexpression vectors in combination with or without ZEB1-specific overexpression vectors.

Finally, in order to further investigate the role of TRIM9-ZEB1 interaction in the malignant phenotype of esophageal cancer cells, ZEB1-specific overexpression vectors were utilized, and their regulatory function on ZEB1 mRNA and protein expression was validated (Figure 6(a)). Then, the modulatory effects of single or combinatory transfection of ZEB1 and TRIM9 overexpression vectors on the expression of stem-like cell biomarkers (ALDH1 and CD133) were evaluated. And the results indicated that the combinatory transfection of ZEB1 overexpression vectors abrogated the inhibitory effects of TRIM9 overexpression transfection on ALDH1/CD133 mRNA and protein levels (Figure 6(b)). In addition, cell migration/invasion assay also showed that ZEB1 overexpression vector cotransfection in KYSE-30 cells could alleviate the effects of TRIM9 overexpression vector transfection on tumor cell migration and invasion (Figures 6(c) and 6(d)). Subsequent mRNA detection of EMT biomarkers confirmed that cotransfection of ZEB1 significantly reversed the promotive effects of TRIM9 overexpression vectors on the epithelial phenotype (Figure 6(e)). The above results suggested that TRIM9-ZEB1 played an interesting role in the pathogenesis and progression of esophageal cancer cells, which could be a potential therapeutic intervention target.

## 4. Discussion

In this study, we demonstrated that a TRIM9 expression decrease was characteristic of esophageal cancer cells. Subsequent functional experiments provided novel evidence that TRIM9 exhibited tumor suppressive effects through the interaction with ZEB1. Upon protein-protein interaction with ZEB1 protein, TRIM9 notably accelerated ZEB1 protein degradation velocity through the UPP pathway. The evidence provided in this study was consistent with previous research conducted by Mishima et al. Through the bisulfite NGS method on breast cancer cell lines and clinical samples, the results of their study demonstrated that high TRIM9 methylation status was characteristic in over 90% of breast cancer cell lines and in over 60% of breast tumor samples. And high TRIM9 promoter methylation status was correlated with significantly suppressed TRIM9 mRNA levels, which could be a potential biomarker for breast cancer [18].

The conclusion of this research suggested that aberrant methylation of TRIM9 might also account for the TRIM9 expression suppression in esophageal cancer, which deserved further investigation. Additionally, future evaluation should also be performed to determine whether demethylation agents might play a role in reversing the suppressive state of TRIM9 expression. Meanwhile, an interesting observation was also reported in uterine leiomyoma, suggesting that TRIM9 overexpression enhanced cell proliferation and antiapoptosis by boosting the activities of subsequent prosurvival signal pathways including survivin and NF-*κ*B [23]. The results of this study indicated that the aberrant TRIM9 expression as well as the TRIM9-related molecular regulatory network might also be tumor-specific, and more expanded research should be performed to fully understand the pattern of TRIM9 expression and its significance in different malignancies.

In addition, it is well known that ZEB1/2 belongs to the zfh family of transcription factors, and ZEB1 can interact with Smad/p300 protein via multiple binding domains of its protein. Previous studies indicated the regulatory role of ZEB1 in the EMT process through the modulation of several related genes. More functional research clarified that ZEB1 participated in the cell polarity factor and basement membrane synthesis inhibition but promoted the matrix metalloprotease level; therefore, ZEB1 greatly promoted basement membrane remodeling and subsequent cancer cell invasion [24]. Furthermore, evidence also indicated that ZEB1 was involved in tumorigenesis and tumor expansion, as a ZEB1 knockout model resulted in diminished acinar-ductal metaplasia [25], which suggested ZEB1's involvement in pancreatic tumorigenesis. Our study demonstrated clues that the TRIM9-ZEB1 regulatory network might possess potential value in future treatment strategy development for esophageal cancer.

It is worth mentioning that future consolidation studies are still required to consolidate the role of TRIM9 in esophageal cancer patients. And TRIM9/ZEB1 knockout *in vitro* or *in vivo* models also will be of value in validating the role of the TRIM9-ZEB1 axis in esophageal cancer pathogenesis and progression.

## 5. Conclusion

In this study, we reported for the first time that TRIM9 was suppressed in esophageal cancer patients. And TRIM9 inhibited esophageal cancer tumor expansion, invasion, and metastasis through interaction with ZEB1, which accelerated its protein degradation through the UPP pathway. Our study demonstrated that the TRIM9-ZEB1 axis might be of value for future esophageal cancer therapy development.

## Data Availability

The datasets used and/or analyzed during the current study are available from the corresponding authors on reasonable request.

## Supplementary Materials

Supplementary Materials Supplementary Table 1: primers used in this research.
