# Correction to “TRIM9 Interacts with ZEB1 to Suppress Esophageal Cancer by Promoting ZEB1 Protein Degradation via the UPP Pathway”

**DOI:** 10.1155/bmri/9847531

**Published:** 2025-10-21

Z. Lin, J. Huang, L. Zhu, et al., “TRIM9 Interacts With ZEB1 to Suppress Esophageal Cancer by Promoting ZEB1 Protein Degradation via the UPP Pathway,” *BioMed Research International* 2023 (2023): 2942402, https://doi.org/10.1155/2023/2942402

In the article, the authors have identified errors in the figures:

• In Figure 2f, in the first row (0 h) for the wound healing experiments on KYSE‐410, the images of sh1 and sh2 are duplicated.
• In Figure 3e, the CD44 and TRIM9 western blots are duplicated.
• In Figure 4d, in the row corresponding to ZEB1, the first and second images are duplicated.

These errors were inadvertently introduced during the production process, and the correct images were present during the peer review of the manuscript. The correct figures are shown as follows:

Figure 2TRIM9 suppression aggravates malignancy of esophageal cancer cells. (a) mRNA and protein expression of TRIM9 modulated by TRIM9‐specific shRNAs (sh1/sh2) and TRIM9 overexpression plasmid in KYSE‐410 and KYSE‐30 esophageal cancer cell lines. (b, c) Functional tests on tumor cell migration and invasion capability of KYSE‐30/KYSE‐410 cell lines transfected with TRIM9 overexpression vectors or TRIM9‐specific shRNAs. Percentage of cell migration and invasion of each cell group was calculated and statistically compared. (d–g) Wound healing experiment on KYSE‐30/KYSE‐410 cell lines transfected with TRIM9‐specific overexpression vectors and TRIM9‐specific shRNAs, respectively. Percentage of migrated tumor cells in the two cell groups was further calculated and statistically compared. (h, i) Protein and mRNA EMT biomarkers (E‐cadherin, N‐cadherin, and vimentin) were detected by WB/qRT‐PCR experiments. Tumor cell group was transfected with TRIM9‐specific shRNAs or overexpression vectors.

**Figure:**
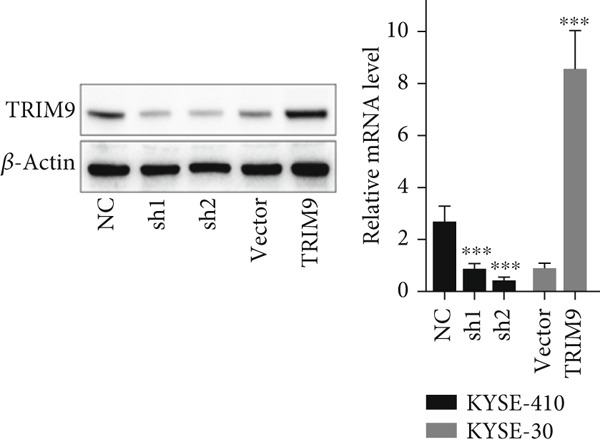
(a)

**Figure:**
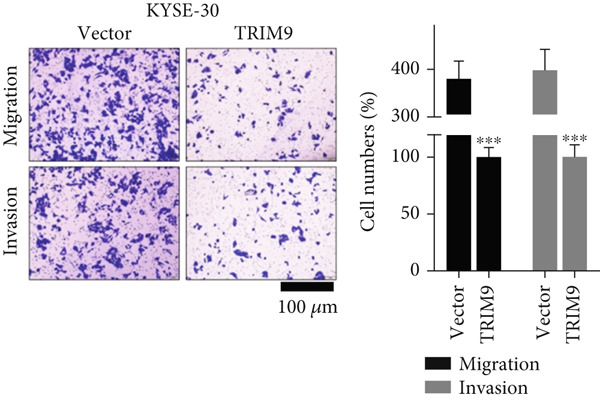
(b)

**Figure:**
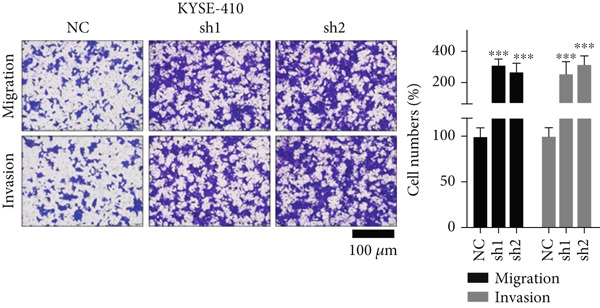
(c)

**Figure:**
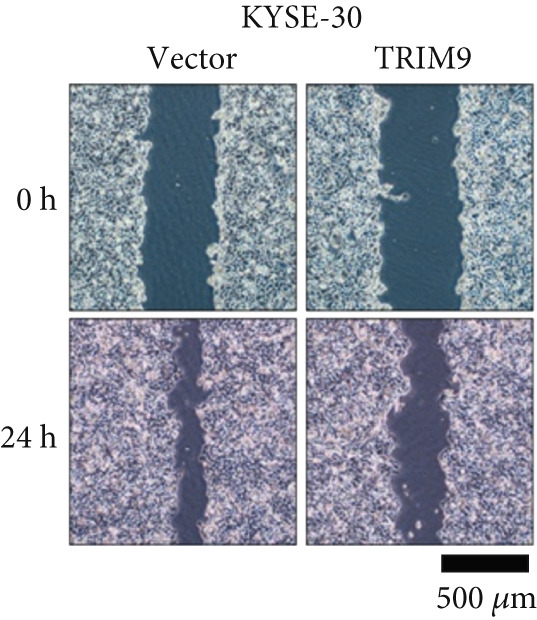
(d)

**Figure:**
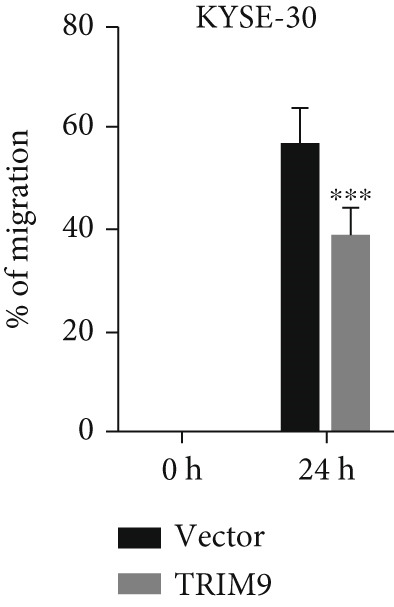
(e)

**Figure:**
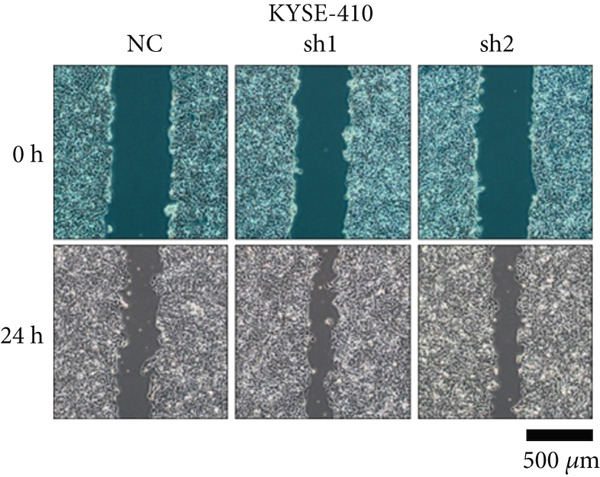
(f)

**Figure:**
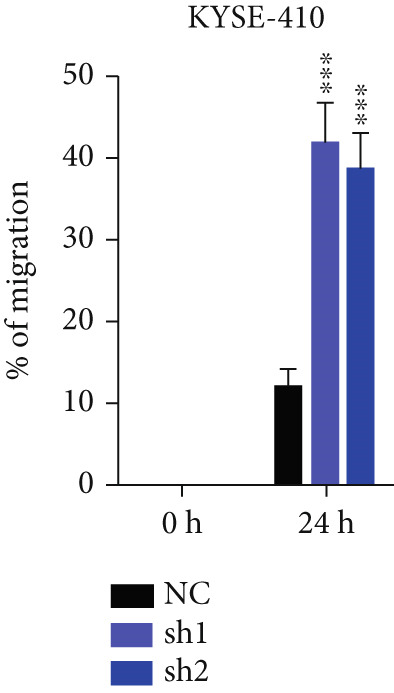
(g)

**Figure:**
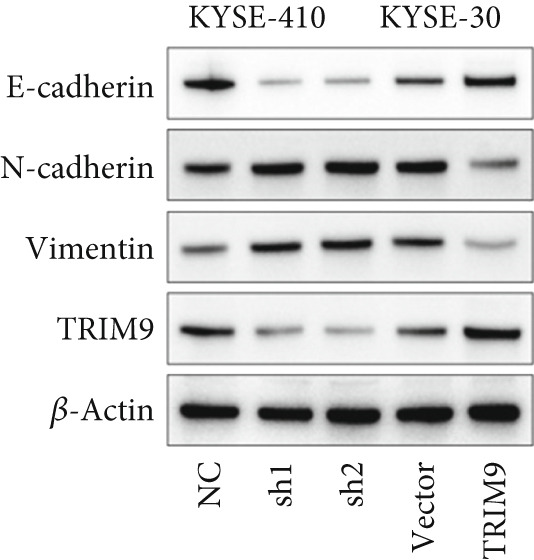
(h)

**Figure:**
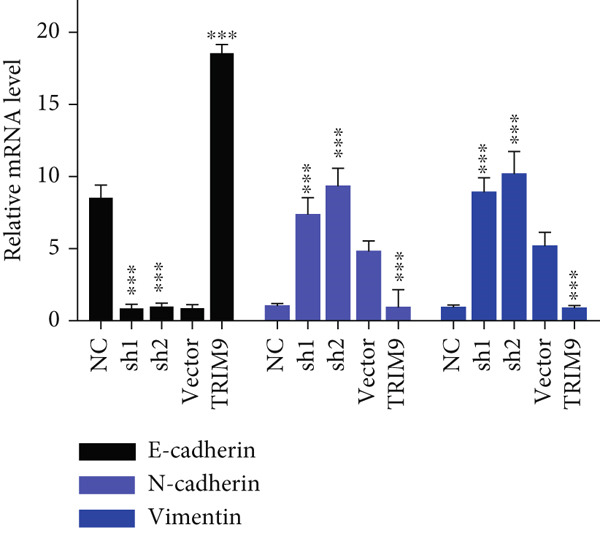
(i)

Figure 3Influence of TRIM9 on chemoresistance of KYSE‐410 cells. (a, b) IC_50_ level detection of chemoagents including fluorouracil and cisplatin treated on esophageal cancer cells. KYSE‐410 and KYSE‐30 cell line groups were treated with TRIM9‐specific shRNAs and overexpression vectors. (c, d) Chemoresistance evaluation of KYSE‐30/KYSE‐410 cell line groups under the treatment of 5‐FU, cisplatin, or paclitaxel. Cancer cells in each of the groups were separately transfected with TRIM9‐specific shRNAs and overexpression vectors/control vectors. (e, f) WB/quantitative RT‐PCR detection of cancer stem–like cell biomarker (CD133/CD44) levels. Tumor cells in each group were separately treated with TRIM9‐specific shRNAs or TRIM9‐specific overexpression vectors and negative control vectors.

**Figure:**
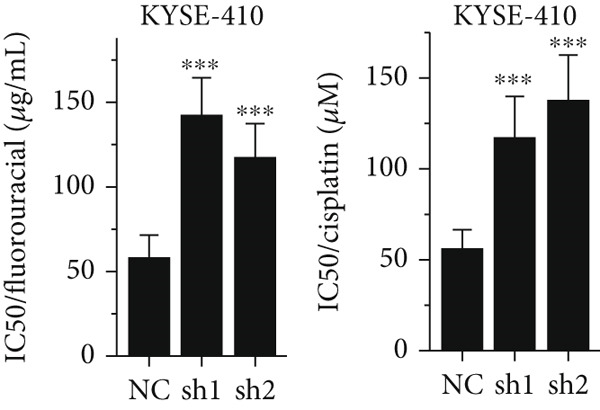
(a)

**Figure:**
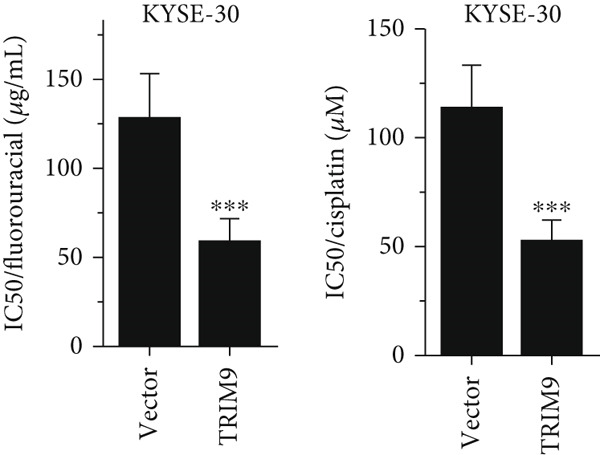
(b)

**Figure:**
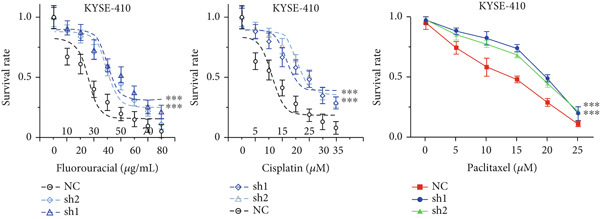
(c)

**Figure:**
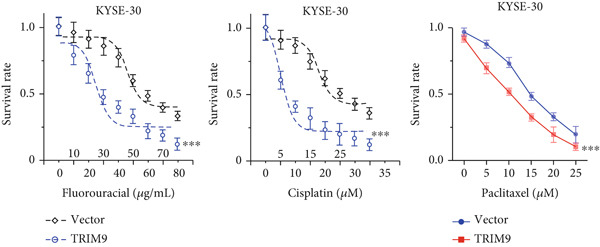
(d)

**Figure:**
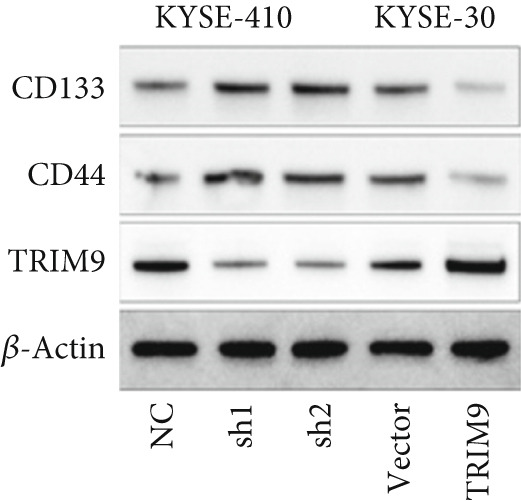
(e)

**Figure:**
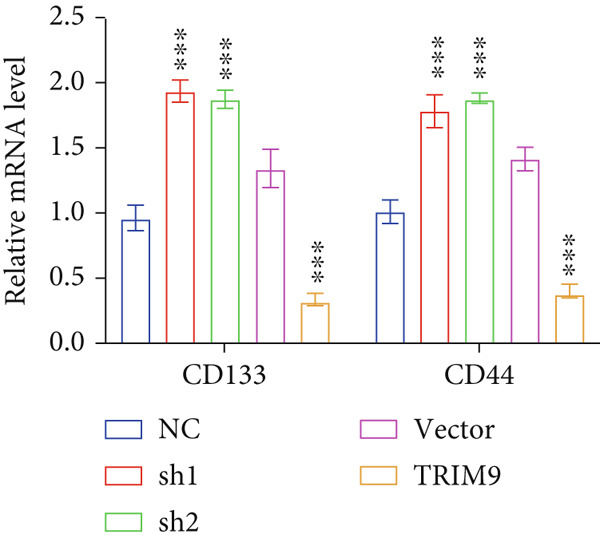
(f)

Figure 4TRIM9 inhibits esophageal cancer cell migration and invasion by regulating ZEB1 via the ubiquitin–proteasome pathway. (a, b) Association of the ZEB1 and TRIM9 protein or mRNA expression levels in esophageal cancer samples. Linear regression analysis was performed to examine statistical significance. (c) WB and quantitative RT‐PCR detection of ZEB1 protein and mRNA levels in KYSE‐410 and KYSE‐30 cell lines. Each group of cancer cells was separately transfected with TRIM9‐specific shRNAs or TRIM9‐specific overexpression vectors and negative control vectors. (d) TRIM9 and ZEB1 protein level detection via WB experiments on esophageal cancer cell groups separately transfected with TRIM9‐specific overexpression vectors or control vectors, in combination with or without treatment of UPP inhibitors (CQ and MG132). (e) Protein level quantification by WB experiments of FLAG‐tagged TRIM9 and ZEB1 in HEK‐293T cells. (f, g) Coimmunoprecipitation assay on the interaction of TRIM9 with ZEB1 in KYSE‐410 and HEK‐293T cell lines. (h) Immunofluorescence assay and colocalization ratio quantification were performed to investigate the colocalization of TRIM9 and ZEB1 in KYSE‐30 and KYSE‐410 cell lines.

**Figure:**
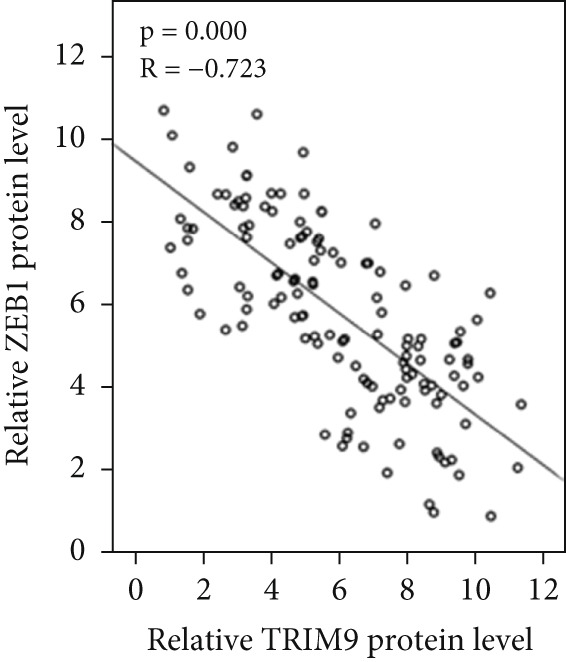
(a)

**Figure:**
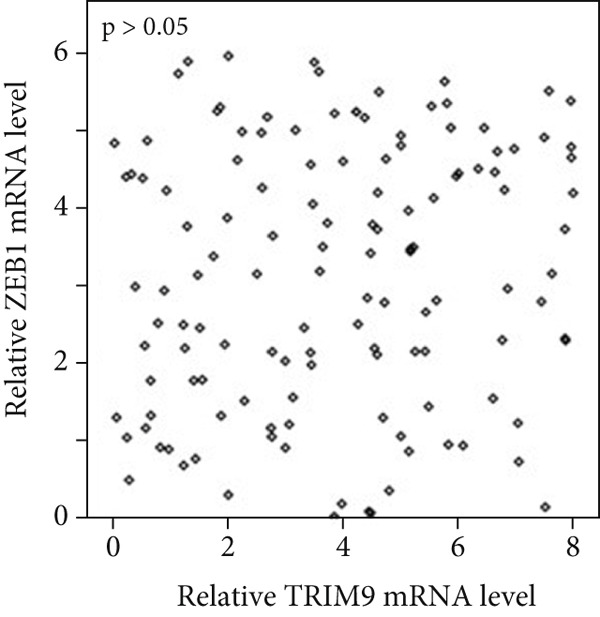
(b)

**Figure:**
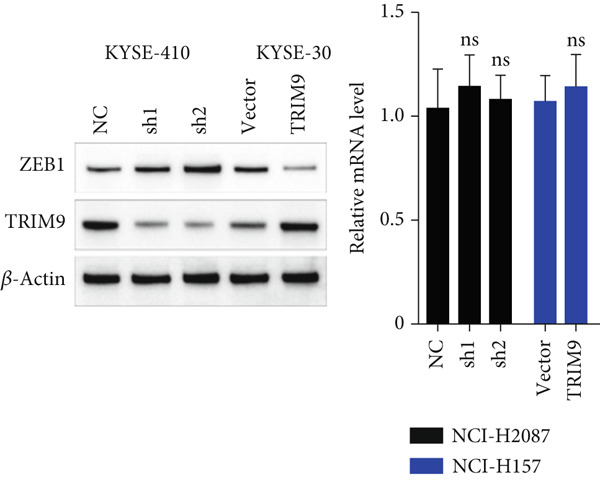
(c)

**Figure:**
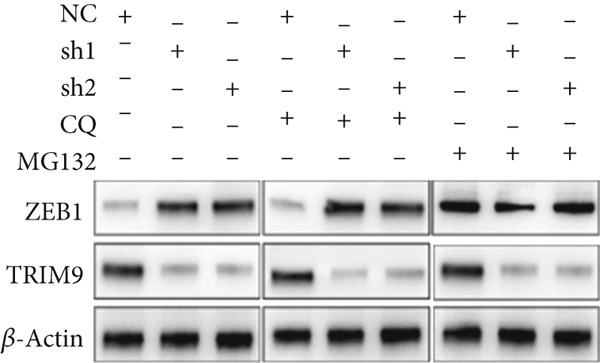
(d)

**Figure:**
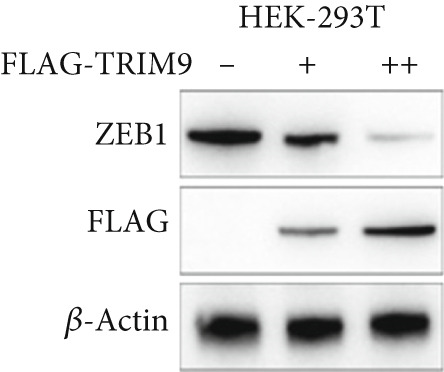
(e)

**Figure:**
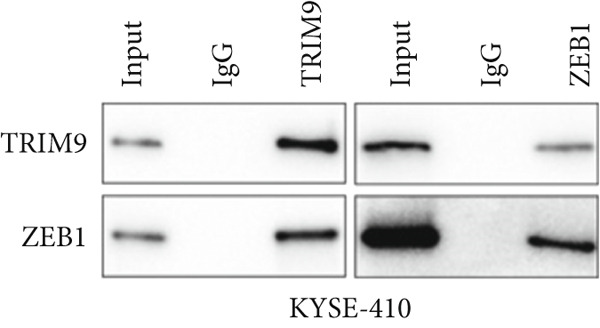
(f)

**Figure:**
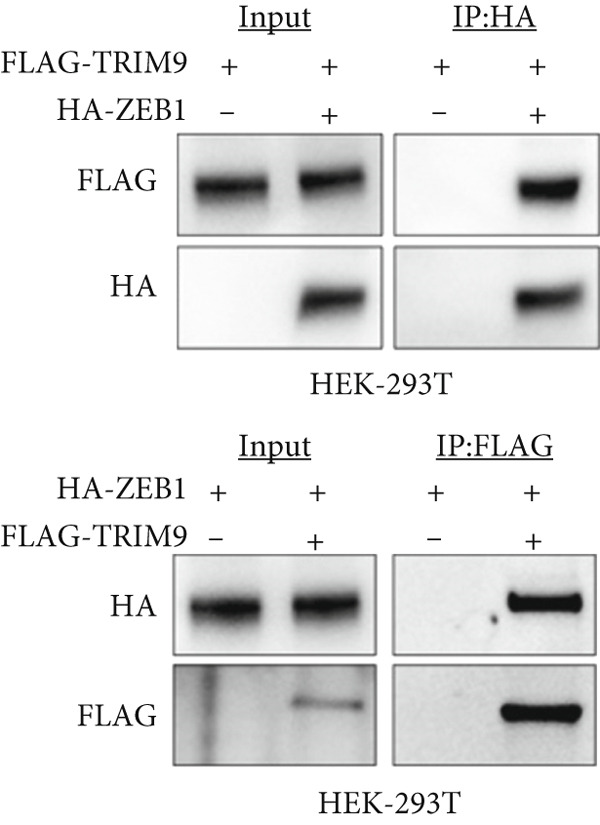
(g)

**Figure:**
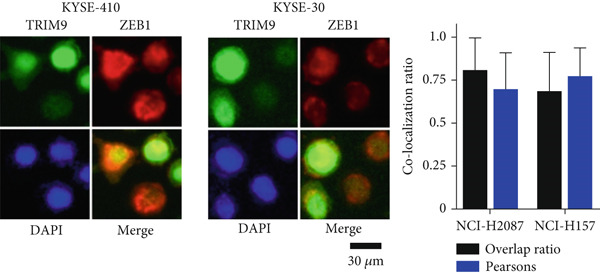
(h)

We apologize for these errors.

