# Supplementary material for: TRIM9 Interacts with ZEB1 to Suppress Esophageal Cancer by Promoting ZEB1 Protein Degradation via the UPP Pathway
Source: Biomed Res Int. 2023 Apr 20;2023:2942402. doi: 10.1155/2023/2942402 (PMC10139803; doi:10.1155/2023/2942402)
Supplement: Supplementary Materials — Supplementary Table 1: primers used in this research. [file 2942402.f1.docx]

| **Supplementary Table 1 Primers used in this research** | | |
| --- | --- | --- |
| Gene | Forward/Reverse | Sequence |
| TRIM9 | Forward | 5’- GTGTGCTACCAGTGCTTGGA |
| TRIM9 | Reverse | 5’- TGTCTGACAGTCCGTTCAGC |
| ZEB1 | Forward | 5’- GGCATACACCTACTCAACTACGG |
| ZEB1 | Reverse | 5’- TGGGCGGTGTAGAATCAGAGTC |
| ALDH1 | Forward | 5’- GGAATACCGTGGTTGTCAAGCC |
| ALDH1 | Reverse | 5’- CCAGGGACAATGTTTACCACGC |
| CD44 | Forward | 5’- CCAGAAGGAACAGTGGTTTGGC |
| CD44 | Reverse | 5’- ACTGTCCTCTGGGCTTGGTGTT |
| E-Cadherin | Forward | 5’- GCCTCCTGAAAAGAGAGTGGAAG |
| E-Cadherin | Reverse | 5’- TGGCAGTGTCTCTCCAAATCCG |
| N-Cadherin | Forward | 5’- CCTCCAGAGTTTACTGCCATGAC |
| N-Cadherin | Reverse | 5’- GTAGGATCTCCGCCACTGATTC |
| Vimentin | Forward | 5’- AGGCAAAGCAGGAGTCCACTGA |
| Vimentin | Reverse | 5’ - ATCTGGCGTTCCAGGGACTCAT |
| GAPDH | Forward | 5’ -GTCCATGCCATCACTGCCAC |
| GAPDH | Reverse | 5’ -AAGGCTGTGGGCAAGGTCAT |
